# *In vitro* imaging of bacteria using ^18^F-fluorodeoxyglucose micro positron emission tomography

**DOI:** 10.1038/s41598-017-05403-z

**Published:** 2017-07-10

**Authors:** Marjolein Heuker, Jürgen W. A. Sijbesma, Rocío Aguilar Suárez, Johan R. de Jong, Hendrikus H. Boersma, Gert Luurtsema, Philip H. Elsinga, Andor W. J. M. Glaudemans, Gooitzen M. van Dam, Jan Maarten van Dijl, Riemer H. J. A. Slart, Marleen van Oosten

**Affiliations:** 1 Department of Medical Microbiology, University of Groningen, University Medical Center Groningen, Hanzeplein 1, PO Box 30001, 9700 RB Groningen, The Netherlands; 2 Department of Nuclear Medicine and Molecular Imaging, University of Groningen, University Medical Center Groningen, Hanzeplein 1, PO Box 30001, 9700 RB Groningen, The Netherlands; 3 Department of Clinical Pharmacy and Pharmacology, University of Groningen, University Medical Center Groningen, Hanzeplein 1, PO Box 30001, 9700 RB Groningen, The Netherlands; 4 Department of Surgery, Division of Surgical Oncology and Intensive Care, University of Groningen, University Medical Center Groningen, Hanzeplein 1, PO Box 30001, 9700 RB Groningen, The Netherlands; 5 Department of Biomedical Photonic Imaging, University of Twente, PO Box 217, 7500 AE Enschede, The Netherlands

**Keywords:** Bacteriology, Infectious-disease diagnostics, Bacterial infection

## Abstract

Positron emission tomography (PET) with fluorine-18-fluorodeoxyglucose (^18^F-FDG) can be applied to detect infection and inflammation. However, it was so far not known to what extent bacterial pathogens may contribute to the PET signal. Therefore, we investigated whether clinical isolates of frequently encountered bacterial pathogens take up ^18^F-FDG *in vitro*, and whether FDG inhibits bacterial growth as previously shown for 2-deoxy-glucose. 22 isolates of Gram-positive and Gram-negative bacterial pathogens implicated in fever and inflammation were incubated with ^18^F-FDG and uptake of ^18^F-FDG was assessed by gamma-counting and µPET imaging. Possible growth inhibition by FDG was assayed with *Staphylococcus aureus* and the Gram-positive model bacterium *Bacillus subtilis*. The results show that all tested isolates accumulated ^18^F-FDG actively. Further, ^18^F-FDG uptake was hampered in *B*. *subtilis pts* mutants impaired in glucose uptake. FDG inhibited growth of *S*. *aureus* and *B*. *subtilis* only to minor extents, and this effect was abrogated by *pts* mutations in *B*. *subtilis*. These observations imply that bacteria may contribute to the signals observed in FDG-PET infection imaging *in vivo*. Active bacterial FDG uptake is corroborated by the fact that the *B*. *subtilis* phosphotransferase system is needed for ^18^F-FDG uptake, while *pts* mutations protect against growth inhibition by FDG.

## Introduction

Bacterial infections represent a major problem in hospital and community settings worldwide. In particular, due to increasingly ageing populations, higher rates of organ transplantations and expanding possibilities for chemotherapy, the numbers of immune-compromised patients who are at risk of bacterial infections are steadily increasing. Moreover, this problem is exacerbated by increased use of implanted biomaterials and medical devices, which are particularly susceptible to bacterial contaminations and infection^1^. Another major contributor to the problem of bacterial infections is the rapid increase of resistance to multiple antibiotics^2^. Infections caused by such multidrug-resistant microbes are difficult to treat and responsible for substantial patient morbidity and mortality^2, 3^.

The diagnosis of bacterial infections is nowadays mainly based on a combination of several specific and/or non-specific symptoms, systemic inflammation markers and the detection of pathogens by culturing, mass spectrometry, polymerase chain reaction (PCR) or serology. In the ideal situation, the causative agent is identified by culturing, but this takes time and is often challenging since sites of infection may be poorly accessible. Also, there is always a risk of contaminations by other bacteria not related to the actual infection resulting in false-positive culture outcomes. Since these challenges often lead to blind management of bacterial infections, there is an urgent need for diagnostic tools that allow real-time detection of infecting bacteria in the patient, for example by infection imaging^1, 4, 5^.

Although several promising approaches for the direct imaging of infecting bacteria have been proposed and explored, with the exception of ^99m^Technetium-ciprofloxacin (^99m^Tc-ciprofloxacin Infecton®)^6^, there are currently no bacteria-specific imaging approaches in clinical use^1, 4^. A clinically applied alternative is positron emission tomography (PET) with fluorine-18-fluorodeoxyglucose (^18^F-FDG), which can serve as a diagnostic tool for localizing infections, inflammatory conditions and unknown origins of fever^7, 8^. In this case, it is generally believed that the infection-specific signal originates from increased glucose uptake by cells involved in the inflammatory process, such as leukocytes, monocytes, lymphocytes, giant cells and macrophages^9^. Importantly, ^18^F-FDG-PET is safe, sensitive, easy and rapid to use^3, 10–12^, and it is potentially applicable in the monitoring of treatment^13^. Furthermore, the view that ^18^F-FDG-PET imaging enables the visualization of bacterial infections and evaluation of antimicrobial therapy has been confirmed in animal experiments and clinical studies^14–18^. On the other hand, diagnosing infections with ^18^F-FDG-PET still remains challenging due to the fact that, for instance, malignancies, sterile inflammation and physiological wound healing, may result in similarly high levels of ^18^F-FDG uptake as infections^9, 15–17^.

An intriguing question that has, so far, received little attention is whether infecting bacteria could potentially contribute to the signal derived in ^18^F-FDG-PET imaging. Specifically, it was not known whether different bacteria can accumulate ^18^F-FDG, and whether the uptake of FDG is potentially toxic for bacteria as is known to be the case for the structurally related glucose analog 2-deoxy-glucose (2-DG)^19^. The aims of this study were, therefore, to determine whether clinical isolates of different bacterial pathogens actively take up ^18^F-FDG *in vitro*, and whether this may lead to impaired bacterial growth.

## Material and Methods

### Bacteria

Clinical isolates of different bacterial pathogens were collected from the diagnostic laboratory of the Department of Medical Microbiology at the University Medical Center Groningen (UMCG; July-November 2015, 15 isolates derived from 15 different patients, Table 1). Identification of these bacterial isolates was performed using matrix-assisted laser desorption/ionization time-of-flight (MALDI-TOF) mass spectrometry (Brüker, Daltonik GmbH, Leipzig, Germany)^20^. 7 bacterial strains from the American Type Culture Collection (ATCC) were also included in the analyses (Table 1). These different bacteria were chosen to cover the most commonly encountered bacterial pathogens at the UMCG. Bacteria were cultured on blood agar plates (5% sheep blood, Mediaproducts, Groningen, The Netherlands) for 1–3 days at 37 °C.

**Table 1:** Bacterial strains for ^18^F-FDG uptake experiments.

Strain	Origin or American Type Culture Collection strain number	Date of isolation at UMCG
*Citrobacter freundii*	clinical isolate	July 2015
	clinical isolate	November 2015
*Enterococcus faecalis*	ATCC 29212	—
	clinical isolate	November 2015
*Enterococcus faecium*	clinical isolate	July 2015
	clinical isolate	November 2015
*Escherichia coli*	ATCC 25922	—
	clinical isolate	October 2015
*Klebsiella pneumoniae*	ATCC 700721	—
	clinical isolate	November 2015
*Proteus mirabilis*	ATCC 12453	—
	clinical isolate	October 2015
*Pseudomonas aeruginosa*	ATCC 27853	—
	clinical isolate	October 2015
*Staphylococcus aureus*	clinical isolate	July 2015
	clinical isolate	October 2015
*Staphylococcus epidermidis*	ATCC 12228	—
	clinical isolate	November 2015
*Streptococcus pneumoniae*	ATCC 49619	—
	clinical isolate	November 2015
*Streptococcus pyogenes*	clinical isolate	October 2015
	clinical isolate	November 2015

### ^18^F-FDG uptake studies

For ^18^F-FDG uptake studies, *Streptococcus pneumoniae*, *Enterococcus faecalis* and *Enterococcus faecium* were cultured in brain-heart infusion (BHI), while all other bacteria were cultured in tryptic soy broth (TSB). All bacteria were grown overnight at 37 °C under constant agitation (250 rpm), with the exception of *S*. *pneumoniae* which was grown in standing culture at 5% CO_2_. The next day, fresh cultures were prepared by dilution of overnight cultures in 9 mL TSB or BHI, and growth was continued up to an optical density (OD) of 1–5 McFarland units. Heat-killed *Staphylococcus aureus* and *Escherichia coli* were obtained by incubating the respective cultures for 30 min at 99 °C. To assess ^18^F-FDG uptake, the bacterial cultures were incubated with 5–10 megabecquerel (MBq) ^18^F-FDG dissolved in 0.5 mL NaCl (0.9%) at 37 °C for 5 min. Subsequently, 2 mL samples were ‘washed’ twice by centrifugation and resuspension of the bacterial pellets in phosphate buffered saline (PBS). To image ^18^F-FDG uptake, the bacteria were pelleted by centrifugation, and four Eppendorf tubes containing bacterial pellets were positioned at the center of the ring system of a micro-PET (µPET) Focus 220 scanner (Siemens Medical Solutions, TN, US) with the bacterial pellets in the field of view. Images were recorded in 30 min and analyzed with the AMIDE software package (Amide’s a Medical Imaging Data Examiner). To quantify ^18^F-FDG uptake, the bacterial pellets were placed in a calibrated gamma-counter (CompuGamma CS1282, LKB-Wallac, Turku, Finland). The radioactivity in each sample was measured, corrected for background and converted into kilobecquerel (kBq). Each ^18^F-FDG uptake experiment was performed thrice and all quantifications were executed in quadruple with two different clinical isolates per tested bacterial species. Numbers of colony forming units (CFUs) in different samples were determined in duplicate by resuspension of the bacterial pellets, serial dilution of the resuspended bacteria, and plating on blood agar containing 5% sheep blood. Only in case of *Proteus mirabilis*, MacConkey agar no.3 plates containing crystal violet were used (Mediaproducts, Groningen, The Netherlands). CFUs of bacteria not incubated with ^18^F-FDG were determined as a control to evaluate the effect of ^18^F-FDG on bacterial survival.

### Time course analysis of ^18^F-FDG uptake

Clinical isolates of *S*. *aureus* and *E*. *coli* were grown overnight in TSB as indicated above, and the *Bacillus subtilis* type strain 168 (Table 2) was grown overnight in Lysogeny Broth (LB) at 37 °C under constant agitation (250 rpm). The next day, the bacteria were diluted 1:20 in 12 mL of the respective growth media and grown for ~2.5 h up to an OD of ~2 McFarland (37 °C, 250 rpm). Subsequently, 5–7 MBq ^18^F-FDG (dissolved in 0.5 mL of 0.9% NaCl) was added to the bacterial cultures. Cultures were incubated at 37 °C after the addition of ^18^F-FDG and culture aliquots of 2 mL were withdrawn at 0, 15, 30, 45 and 60 min. To stop ^18^F-FDG uptake, each culture aliquot was washed twice by centrifugation and resuspension of the bacterial pellets in PBS. Lastly, the bacteria were pelleted by centrifugation and ^18^F-FDG uptake in the pellets was measured with a calibrated CompuGamma CS1282 gamma-counter. CFUs were determined at t = 0, t = 30 and t = 60 min by resuspension of the pellets, serial dilution and plating on blood agar as described above. Heat-killed *S*. *aureus* and *E*. *coli* (30 min, 99 °C) served as negative controls. Uptake of ^18^F-FDG over time was evaluated in duplicate.

**Table 2:** Laboratory strains used for growth in the presence of FDG.

Strain	Genotype	References
*S*. *aureus* HG001	*rsbU* repaired, *tcaR*	(33)
*B*. *subtilis* 168	*trpC2*	Laboratory Collection
*B*. *subtilis* QB5435	*trpC2*, *ptsG*::cat, Cm^R^	(34)
*B*. *subtilis* GP864	*trpC2 ptsI::*Em^R^	(35)

Cm^R^, Chloramphenicol resistant; Em^R^, Erythromycin resistant.

### Growth of *S. aureus* and *B. subtilis* in the presence of non-labeled FDG

To assess the toxicity of FDG, the laboratory strain *S*. *aureus* HG001 and different *B*. *subtilis* strains (Table 2) were grown overnight in TSB or LB, respectively. Where appropriate the media were supplemented with chloramphenicol or erythromycin at a concentration of 5 µg/mL. The overnight cultures were, in triplicate, diluted 1:50 in 100 µL fresh TSB or LB in 96-well microtiter plates, and the plates were incubated for 3 h at 37 °C with vigorous agitation (800 rpm). Lastly, each culture was again diluted 1:50 in 100 µL of the respective growth medium supplemented with 2 µg/mL, 20 µg/mL, 200 µg/mL or 2 mg/mL non-radioactive FDG, or without FDG. Cultures were incubated at 37 °C in a Biotek synergy 2 plate reader with shaking and the OD at 600 nm (OD_600_) was recorded at 10 min intervals. Each growth experiment was performed at least twice.

### Statistical analyses

Kruskal Wallis and Mann-Whitney U tests were performed using IBM SPSS Statistics 23. P-values of 0.05 or less were considered significant.

## Results

### *In vitro* uptake of ^18^F-FDG

**Figure 1:**
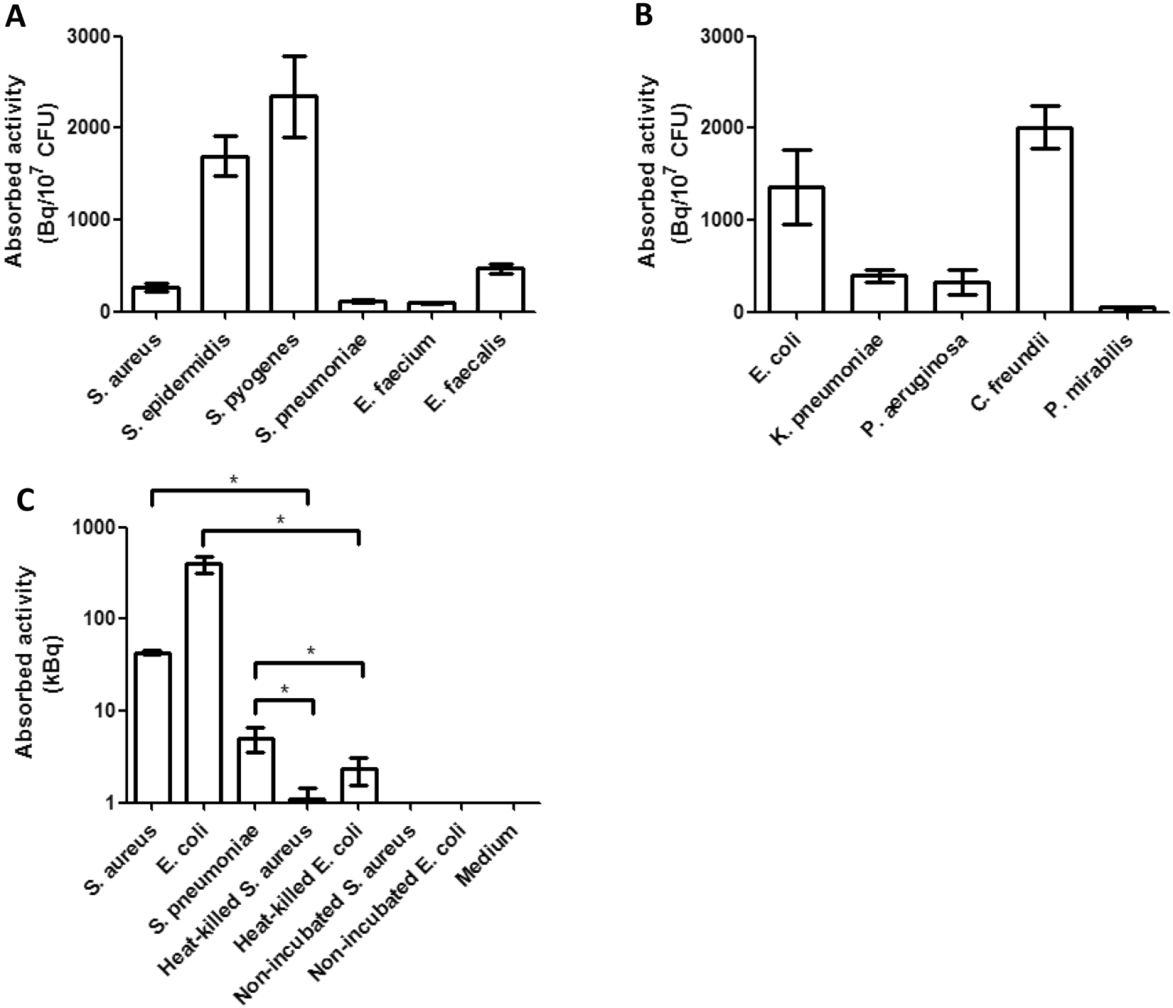
*In vitro* uptake of ^18^F-FDG by different bacteria. The mean uptake of ^18^F-FDG by clinical isolates of different Gram-positive (**A**) and Gram-negative (**B**) bacteria was expressed as absorbed activity in Bq/10^7^ CFUs. (**C**) Total uptake in kBq of ^18^F-FDG by *S*. *aureus*, *E*. *coli* or *S*. *pneumoniae* in comparison to heat-killed bacteria, bacteria not incubated with ^18^F-FDG, and medium controls. The indicated standard deviations include the variations introduced by the use of at least two different isolates per tested species. Note that the detection limit for a bacterial species will lie between the nonspecifically absorbed activity measured for heat-killed bacteria, such as *E*. *coli* and *S*. *aureus*, and the lowest level of ^18^F-FDG uptake by the living bacteria, such as *S*. *pneumoniae*. This implies that under the tested conditions, the detection limit is ~2.4 kBq. *p ≤ 0.01. Values represent mean ± SEM (n = 8).

To evaluate whether bacterial pathogens are capable of active uptake of ^18^F-FDG, a range of different Gram-positive and Gram-negative bacteria including mostly clinical isolates were incubated with ^18^F-FDG, and uptake was determined with a calibrated gamma-counter. The ^18^F-FDG PET signals were quantified and the absorbed activity per CFU was determined as shown in Fig. 1(A and B). All bacterial isolates showed significant ^18^F-FDG uptake. No major differences were observed for most of the investigated Gram-positive and Gram-negative bacteria (p = 0.60; not shown). Nonetheless, statistically significant differences in ^18^F-FDG uptake were observed for different bacterial species (p < 0.01) as illustrated in Fig. 1. The Gram-positive bacterium *Streptococcus pyogenes* displayed overall the highest uptake activity per 10^7^ CFUs (Fig. 1A and B). *E*. *coli* and *Citrobacter freundii* showed the highest ^18^F-FDG uptake amongst the tested Gram-negative species (Fig. 1B). In contrast, *S*. *pneumoniae*, *E*. *faecium* and *P*. *mirabilis* showed relatively low ^18^F-FDG uptake, but as shown for *S*. *pneumoniae* the ^18^F-FDG uptake was still significantly higher than the negative controls (p = 0.01; Fig. 1C). Importantly, other living bacteria, such as *S*. *aureus* and *E*. *coli* accumulated significantly higher ^18^F-FDG levels than the respective heat-killed bacteria, bacteria incubated in the absence of ^18^F-FDG, or medium controls (p = 0.004).

**Figure 2:**
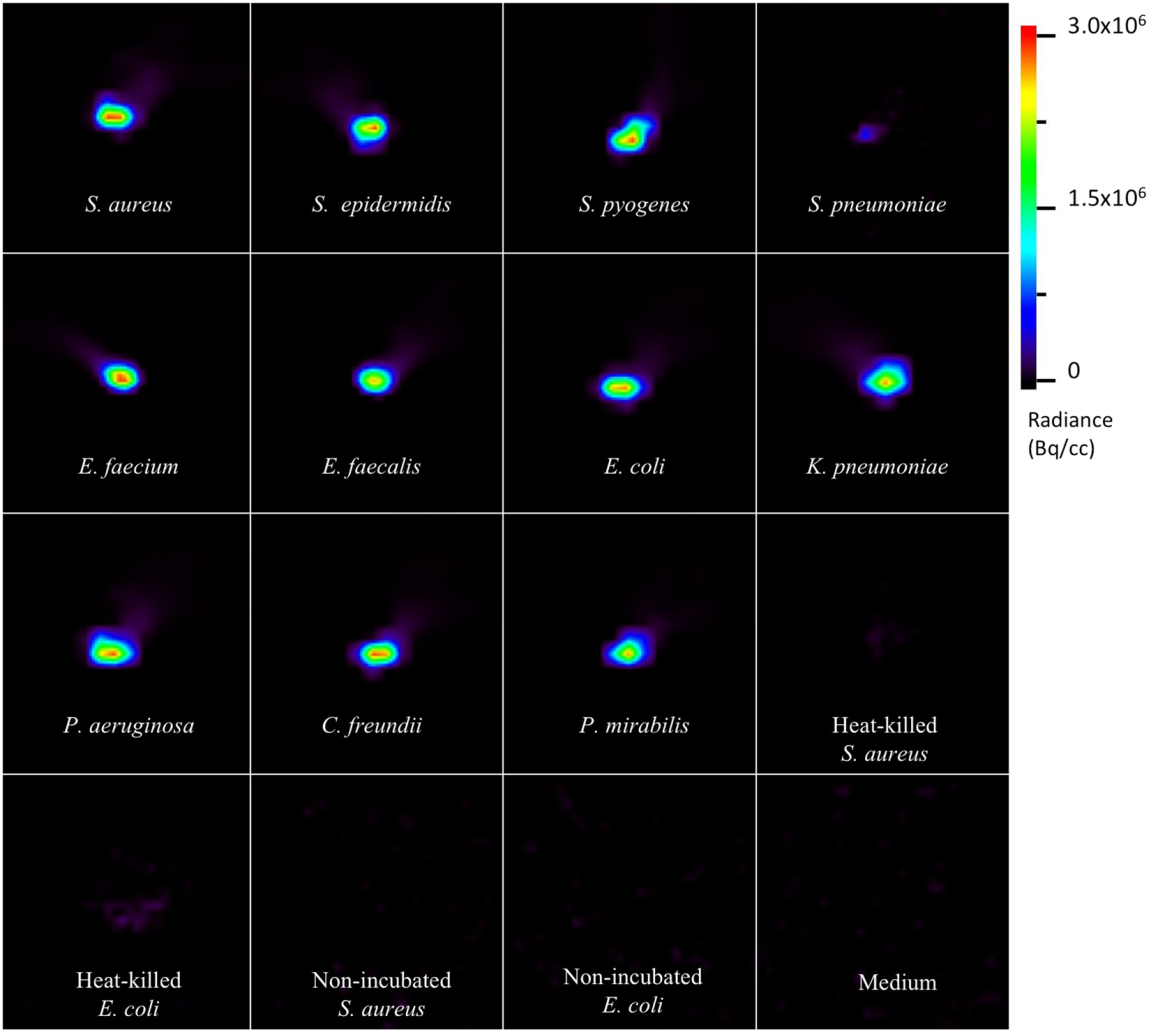
µPET images of ^18^F-FDG uptake by bacterial isolates. Different clinical bacterial isolates were grown to an OD of 1–5 McFarland units in 9 mL of growth medium, pelleted and resuspended in the same volume at 15 min before the incubation with 0.5 ml of 5–10 MBq ^18^F-FDG for 5 min. Heat-killed *S*. *aureus* or *E*. *coli* were obtained by incubating the respective cultures for 30 min at 99 °C. As negative controls, *S*. *aureus* or *E*. *coli* cells incubated without added ^18^F-FDG, or with fresh medium were used. All samples were washed twice in PBS and washed bacterial pellets corresponding to 2 mL culture samples were imaged for 30 min with a µPET scanner for a qualitative evaluation of the bacterial ^18^F-FDG uptake. The scale bar indicates the color map range of Radiance in Bq per cubic centimeter (cc).

To evaluate whether bacterial ^18^F-FDG uptake can also be detected by µPET imaging, the ^18^F-FDG uptake by several Gram-negative and Gram-positive clinical isolates was qualitatively assessed with a µPET Focus 220 scanner. Indeed, the resulting µPET images clearly show ^18^F-FDG uptake by the tested bacteria (Fig. 2). *S*. *pneumoniae* showed the least uptake activity, which might relate to the high autolytic activity of this bacterium^21^. As expected, little if any ^18^F-FDG uptake was detectable for heat-killed *S*. *aureus* and *E*. *coli*, as was the case for non-incubated bacteria and medium controls. Notably, there were some apparent differences in the relative signals observed in Figs 1 and 2 for, respectively, the *E*. *faecium* and *E*. *faecalis* samples, and for the *S*. *aureus* and *S*. *epidermidis* samples. This may relate to the fact that counting with the calibrated gamma-counter as in Fig. 1 provides quantitative data on ^18^F-FDG uptake, whereas the µPET scans as in Fig. 2 give semi-quantitative information on ^18^F-FDG uptake. For example, there may have been some variation in the distribution of the pelleted bacteria in the samples that were imaged by µPET scans. Furthermore, the observed variations may have been due, to some extent, to differing numbers of metabolically active bacteria in the samples respectively used for Fig. 1 and 2.

**Figure 3:**
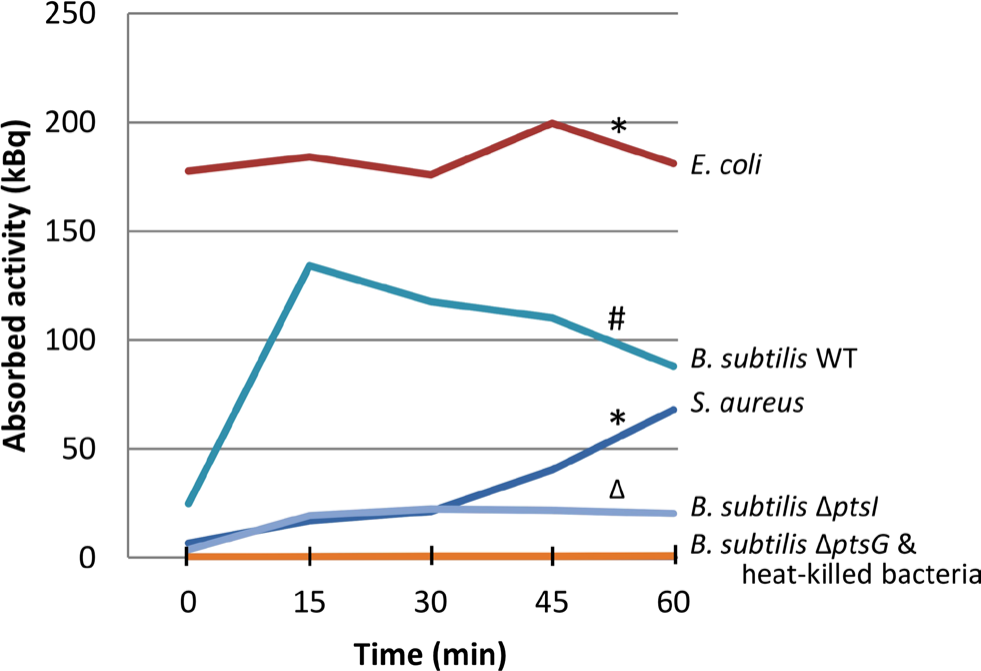
Time course analysis of *in vitro* uptake of ^18^F-FDG. Mean ^18^F-FDG uptake was determined over a period of 60 min for clinical isolates of *S*. *aureus* (red) and *E*. *coli* (dark blue), and for the *B*. *subtilis* type strain 168 (light blue) and its *ptsG* (orange) or *ptsI* mutant (purple) derivatives. Heat-killed bacteria (orange) served as negative controls. Samples were withdrawn at the indicated time points to assess the ^18^F-FDG uptake. *p < 0.01 vs. heat-killed controls; ^#^p < 0.001 vs. both mutants; Δp < 0.001 vs. the Δ*ptsG* mutant.

To approximate the time needed for uptake of ^18^F-FDG, time course experiments were performed with *S*. *aureus* and *E*. *coli*, two bacterial species showing significant differences in ^18^F-FDG uptake (Fig. 1). The results presented in Fig. 3 show indeed significant differences in the rates of uptake by both species, *E*. *coli* reaching saturation almost instantaneously while ^18^F-FDG uptake by *S*. *aureus* was relatively slow. Within 60 min of incubation, *E*. *coli* absorbed significantly more ^18^F-FDG than *S*. *aureus* (p < 0.001), while *E*. *coli* and *S*. *aureus* both showed higher signals than the respective heat-killed controls (p < 0.01).

### Potential mechanism of ^18^F-FDG uptake

In order to assess the possible mechanism of ^18^F-FDG uptake, we applied the Gram-positive model bacterium *B*. *subtilis* for which mutations in the phosphotransferase system (PTS) that facilitates glucose uptake are available^22^. Indeed, *B*. *subtilis ptsI* and *ptsG* deletion strains displayed a strongly impaired uptake of ^18^F-FDG compared to the parental wild-type strain *B*. *subtilis* 168 (p < 0.001; Fig. 3). Of note, the *ptsI* deletion mutant showed a somewhat higher uptake than the *ptsG* deletion mutant (p =  < 0.001), which is in line with the fact that PtsG is the major glucose transporter^23^, while PtsI is involved in the phosphorylation of internalized glucose making its uptake by PtsG irreversible^24^. These findings show that, at least in *B*. *subtilis*, ^18^F-FDG is taken up via the PTS system.

### FDG-impaired bacterial growth

**Figure 4:**
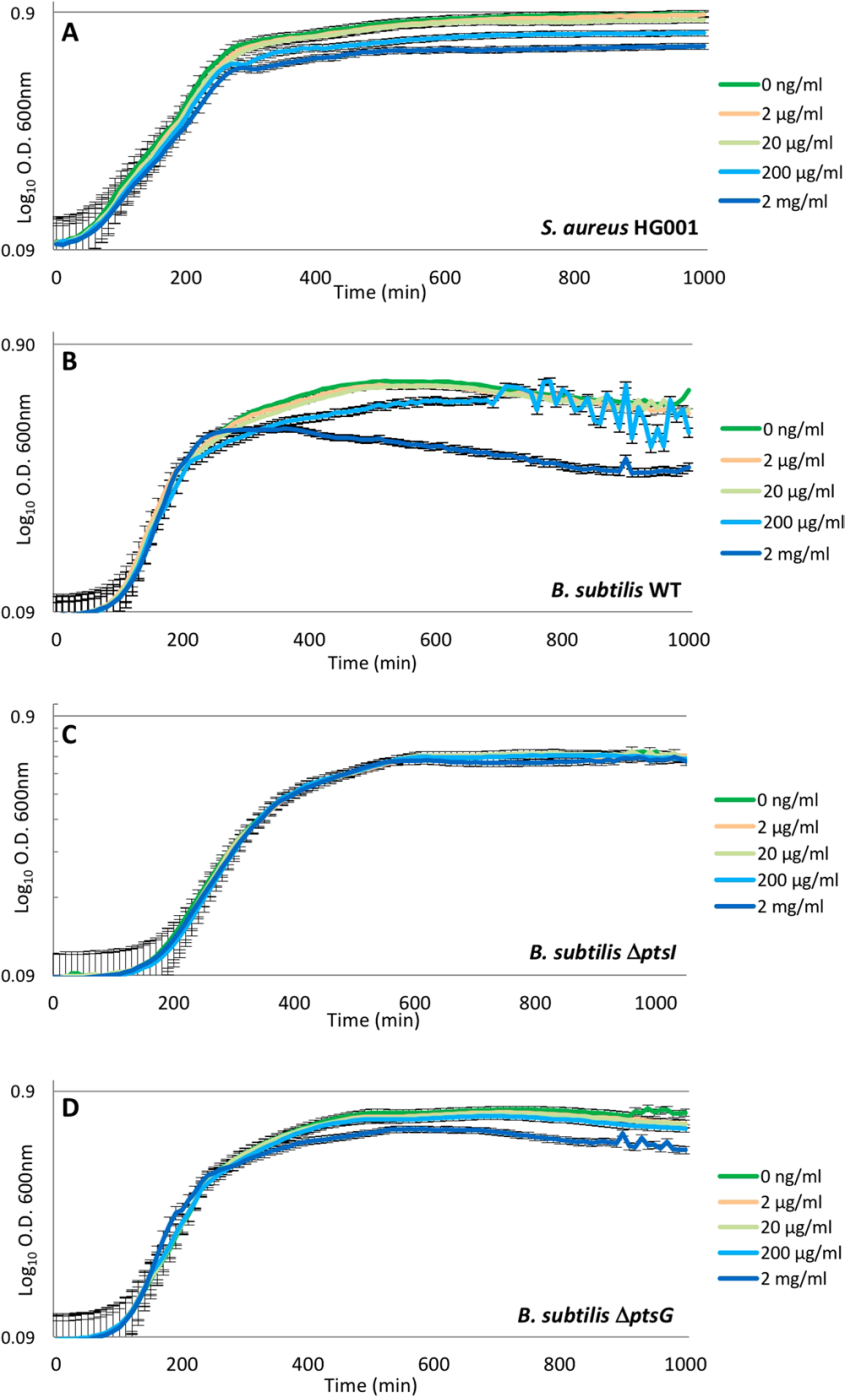
Growth of *S*. *aureus* and *B*. *subtilis* in the presence of non-labeled FDG. *S*. *aureus* HG001 was grown in TSB (**A**), and the *B*. *subtilis* strains 168 (WT), Δ*ptsI* and Δ*ptsG* were grown in LB (**B**,**C** and **D**) with increasing concentrations of non-radiolabeled FDG as indicated in color code. OD_600_ readings were recorded every 10 min.

To test whether FDG could be toxic for bacteria growth experiments were performed with *S*. *aureus* HG001 and *B*. *subtilis* 168, where the growth media were supplemented with increasing concentrations of FDG up to 2 mg/mL. As shown in Fig. 4A, *S*. *aureus* HG001 reached somewhat lowered OD_600_ values in the stationary phase when the medium contained 200 µg/mL FDG or more. Similarly, *B*. *subtilis* reached a lowered optical density in the stationary phase when cultured in the presence of 2 mg/mL FDG (Fig. 4B). This indicates that FDG is mildly toxic for bacteria, such as *S*. *aureus* and *B*. *subtilis*, at high concentrations. To verify this, the *B*. *subtilis ptsI* and *ptsG* deletion mutants were also cultured in the presence of FDG. The results show that both mutations significantly diminished the growth impairment caused by 2 mg/mL FDG (Fig. 4C and D). Together, these findings show that active FDG uptake may be toxic for bacteria, but only at very high concentrations that will not be reached during ^18^F-FDG-PET imaging *in vivo*.

## Discussion

The current study shows for the first time that clinical isolates of many major Gram-positive and Gram-negative bacterial pathogens can actively take up ^18^F-FDG. This is consistent with the previous finding of Weinstein *et al*.^25^, who reported that type strains of *E*. *coli*, *Klebsiella pneumoniae* and *S*. *aureus* can take up this compound. The combined findings imply that bacterial pathogens may contribute to the signal observed in ^18^F-FDG PET imaging of bacterial infections. Furthermore, the observed ^18^F-FDG uptake was, at least in *B*. *subtilis*, shown to be facilitated by the PTS system for glucose uptake. Lastly, our present observations show that FDG may be toxic for bacteria but only at very high doses that exceed the physiological levels during ^18^F-FDG PET imaging about 100- to 1000-fold.

Notably, of all presently tested bacterial species, *S*. *pyogenes* displayed the highest uptake of ^18^F-FDG. This bacterium is notorious for causing necrotizing fasciitis, a rapidly progressive infection of deep layers of the skin and subcutaneous tissues, which requires immediate antibiotic therapy and aggressive surgery^26^. The high uptake levels of ^18^F-FDG suggest that *S*. *pyogenes* may have been the metabolically most active bacterium under the tested conditions. All other bacteria showed lower ^18^F-FDG uptake *in vitro*. In view of the fulminant pathology of invasive *S*. *pyogenes* infections, it is conceivable that this bacterium displays also a high metabolic activity *in vivo*.

In line with the finding that ^18^F-FDG is actively taken up by the investigated bacteria, the absence of the glucose transporter PtsG abrogated FDG uptake by *B*. *subtilis*. The absence of PtsI, also known as Enzyme I of the PTS system^22^, had a strong but less drastic negative effect on ^18^F-FDG uptake suggesting that its contribution to FDG uptake can to some extent be bypassed. Further, our present results show that FDG is toxic for *S*. *aureus* and *B*. *subtilis*, but only at high concentrations that are not reached during ^18^F-FDG PET imaging *in vivo*. Yet, the observed toxicity of FDG is reminiscent of the toxic effects of 2-DG. In the latter case, the PTS system recognizes 2-DG and phosphorylates it into 2-DG-6-phosphate, which is subsequently dephosphorylated by hexose-6-phosphatase. This cycle of 2-DG phosphorylation and de-phosphorylation depletes the bacterial cell of phosphoenolpyruvate and eventually adenosine triphosphate (ATP)^27^. Thus, 2-DG has the ability to de-energize bacteria. This may also be the case for FDG, which would explain the observed growth inhibition at high FDG concentrations. Of note, the growth of *B*. *subtilis ptsG* or *ptsI* deletion mutants was much less affected by FDG than growth of the parental strain, which is consistent with the reduced uptake of ^18^F-FDG.

Currently, there is ample evidence that ^18^F-FDG PET is a very useful diagnostic tool for many infectious indications, including fever of unknown origin, endocarditis, spondylodiscitis, and vascular graft infections^8, 11, 12^. This approach can help in defining the location and extent of infection, thereby providing guidance for biopsy and for therapy follow-up. In general, the detection limit in the imaging of infections by PET will depend on the sensitivity and resolution of the PET camera, on the volume of the bacterial lesion, on the surrounding tissue, and on the target-to-normal tissue (T/N) ratios of radioactivity at sites of infection. Our present study shows that ^18^F-FDG uptake by infecting bacteria potentially contributes to the overall signal detected by PET imaging of bacterial infections. However, it remains difficult to approximate the contribution of the bacterial ^18^F-FDG uptake to the total ^18^F-FDG uptake signal measured during *in vivo* PET imaging of an infection, because the bacterial contribution to the total signal will vary depending on the bacterial species that causes the infection, on the metabolic state of the infecting bacteria, and on the total number of infecting bacteria. Moreover, the inflammatory status of the infected tissue will impact on the total signal, especially because white blood cell migration may vary depending on the extent of inflammation, and also because inflammation may lead to increased blood flow and vascular permeability. Furthermore, the total signal is likely to depend on the particular body site that is infected. Altogether, this means that, in practice, the relative bacterial contribution to the total ^18^F-FDG signal will be different for individual patients and for different types of infections. For example, chronic infections can be visualized with ^18^F-FDG, but the signal contributed by the bacteria themselves may be lower than in acute infections if their metabolic activity is lower, which would result in lower uptake of ^18^F-FDG. In such cases, an option to improve the bacterial signal could be to start imaging at later time points after tracer injection. Clearly, the conditions *in vivo* are likely to be different compared to the conditions applied in our *in vitro* study. It is therefore presently not possible to make reliable calculations of the detection limit for infections *in vivo*.

Lastly, with ^18^F-FDG PET it is not possible to differentiate between sterile inflammation and infection. Also, ^18^F-FDG PET will neither tell us whether bacteria are involved in an infection, nor which bacteria are involved. Specific bacteria-targeted PET tracers are therefore desirable. For example, these could include labeled antibiotics like ^18^F- or ^99m^Tc-ciprofloxacin (Infecton®)^6, 28–31^. A very promising alternative for ^18^F-FDG may be 2-[18 F]-fluorodeoxysorbitol (^18^F-FDS), since sorbitol is a sugar alcohol that is mainly metabolized by the Gram-negative *Enterobacteriaceae*^5, 25^. Another attractive approach appears to be the detection of bacteria with 6-[^18^F]-fluoromaltose (^18^F-FM)^32^, because maltose and maltodextrins are metabolizable by all classes of bacteria, in contrast to mammalian cells. Thus, ^18^F-FM may allow the distinction between bacterial infection and other causes of inflammation. On this basis it is well conceivable that the combined use of ^18^F-FDS and ^18^F-FM will facilitate the detection of a wide spectrum of bacteria causing infections. With the aid of both substrates, it should not only be possible to specifically image infecting bacteria over mammalian cells, but also to discriminate infecting Gram-positive and Gram-negative bacteria. The latter would then guide effective antimicrobial therapy. Yet, despite these evident advantages, no clinical studies on infection imaging with ^18^F-FDS and ^18^F-FM have thus far been reported.

## Conclusion

To date, ^18^F-FDG is one of the few tracers that may be clinically applied in infection imaging. We now show that this tracer can be taken up by a wide range of commonly encountered bacterial pathogens. We therefore hypothesize that bacterial pathogens may also take up ^18^F-FDG *in vivo*, thereby contributing to the FDG-PET signal observed in infection imaging. Future experiments are needed to determine the proportion of ^18^F-FDG that is taken up by infecting bacteria as compared to the ^18^F-FDG taken up by inflammatory cells. This will unveil the significance of our present findings in the clinical context.
