# Publisher Correction: *In vitro* imaging of bacteria using ^18^F-fluorodeoxyglucose micro positron emission tomography

**DOI:** 10.1038/s41598-019-46786-5

**Published:** 2019-07-17

**Authors:** Marjolein Heuker, Jürgen W. A. Sijbesma, Rocío Aguilar Suárez, Johan R. de Jong, Hendrikus H. Boersma, Gert Luurtsema, Philip H. Elsinga, Andor W. J. M. Glaudemans, Gooitzen M. van Dam, Jan Maarten van Dijl, Riemer H. J. A. Slart, Marleen van Oosten

**Affiliations:** 1 Department of Medical Microbiology, University of Groningen, University Medical Center Groningen, Hanzeplein 1, PO Box 30001, 9700 RB Groningen, The Netherlands; 2 Department of Nuclear Medicine and Molecular Imaging, University of Groningen, University Medical Center Groningen, Hanzeplein 1, PO Box 30001, 9700 RB Groningen, The Netherlands; 3 Department of Clinical Pharmacy and Pharmacology, University of Groningen, University Medical Center Groningen, Hanzeplein 1, PO Box 30001, 9700 RB Groningen, The Netherlands; 4 Department of Surgery, Division of Surgical Oncology and Intensive Care, University of Groningen, University Medical Center Groningen, Hanzeplein 1, PO Box 30001, 9700 RB Groningen, The Netherlands; 5 Department of Biomedical Photonic Imaging, University of Twente, PO Box 217, 7500 AE Enschede, The Netherlands

Correction to: *Scientific Reports* 10.1038/s41598-017-05403-z, published online 10 July 2017

In the original version of this Article, the author Rocio Aguilar Suárez was incorrectly indexed. This error has now been corrected.

